# Overexpression of microRNA-367 inhibits angiogenesis in ovarian cancer by downregulating the expression of LPA1

**DOI:** 10.1186/s12935-020-01551-x

**Published:** 2020-10-02

**Authors:** Qingling Zheng, Xin Dai, Wei Fang, Yan Zheng, Jin Zhang, Yanxiang Liu, Donghua Gu

**Affiliations:** 1 Department of Obstetrics and Gynecology, School of Medicine and Nursing Sciences, Huzhou University, Huzhou Central Hospital, Huzhou, 313000 People’s Republic of China; 2 Department of Pathology, The Affiliated Suzhou Science & Technology Town Hospital of Nanjing Medical University, No. 1, Lijiang Road, Huqiu District, Suzhou, 215153 Jiangsu People’s Republic of China; 3 Department of Pathology, Huzhou Central Hospital, Huzhou, 313000 People’s Republic of China

**Keywords:** Ovarian cancer, microRNA-367, Lysophosphatidic acid receptor-1, Proliferation, Invasion, Angiogenesis

## Abstract

**Background:**

Compelling evidences reported the role of microRNAs (miRNAs) in ovarian cancer. However, little was known regarding the molecular mechanism of miR-367 in ovarian cancer. This study intended to investigate the role and regulatory mechanism of miR-367 in ovarian cancer involving lysophosphatidic acid receptor-1 (LPA1).

**Methods:**

Potentially regulatory miRNAs in ovarian cancer were obtained from bioinformatics analysis. RT-qPCR was used to detect miR-367 expression in both ovarian cancer tissues and relevant adjacent normal tissues. Relationship between miR-367 and LPA1 was predicted by miRNA database and further verified using dual luciferase reporter gene assay and RIP. EdU and Transwell assay were used to measure the proliferation and invasion ability of cells. Moreover, tube formation and chick chorioallantois membrane (CAM) assay were performed to determine angiogenesis of human umbilical vein endothelial cells (HUVECs). Finally, the roles of LPA1 in tumor growth was also studied using nude mice xenograft assay.

**Results:**

High expression of LPA1 and low expression of miR-367 were observed in ovarian cancer tissues and cells. Overexpressed miR-367 downregulated LPA1 expression to inhibit proliferation, invasion, and angiogenesis of cancer cells. Low expression of LPA1 suppressed tumor formation and repressed angiogenesis in ovarian in vivo.

**Conclusion:**

All in all, overexpression of miR-367 downregulated LPA1 expression to inhibit ovarian cancer progression, which provided a target for the cancer treatment.

## Background

Ovarian cancer ranks 7th among cancers in women and 8th cause of cancer death [1]. The progression of ovarian cancer requires the co-evolution of neoplastic cells as well as the adjacent microenvironment [2]. It was reported that the main challenge of ovarian cancer treatment was that most patients have advanced disease at the time of diagnosis [3]. Angiogenesis is a complicated process which greatly affects growth, tissue and organ regeneration, and many pathological conditions [4]. Currently, it is reported that angiogenesis is a multi-step process which needs highly modulated endothelial cell behavior [5]. Angiogenesis was reported to be a crucial marker for ovarian cancer development [6]. Thus, it is urgent to develop new strategy for diagnosing ovarian cancer.

MicroRNAs (miRNAs) are a variety of RNAs that could regulate the translation and stability of mRNAs influencing cell differentiation, migration and apoptosis [7]. Besides, miRNAs influence various physiological states [8]. It has been verified that miRNAs are differentially expressed in ovarian cancer and exert functions as both diagnostic and prognostic targets for ovarian cancer treatment [9]. For example, the relationship between paclitaxel sensitivity and miR-367/miR-30a-5p expression was used as novel therapeutic targets for ovarian cancer treatment [10].

The lysophosphatidic acid (LPA) is a crucial signaling molecule due to its widespread presence in biological fluids and its relation to disease conditions including fibrosis and cancer [11]. LPA is an important component of biofilm, an extracellular signal transmitter and intracellular second messenger, it can target endothelial differentiation gene (Edg) family LPA receptors (LPA1, LPA2, and LPA3) and non-Edg family LPA receptors (LPA4, LPA5, and LPA6) to mediate physiological and pathological processes such as angiogenesis, tumor progression, and inflammatory reactions [12]. The expression of LPA2 or LPA3 contributes to the aggressiveness of ovarian cancer, suggesting that targeting the production and action of LPA may be potential to treat ovarian cancer [13]. Further, inhibition of LPA1 has effects on metastasis and metastatic dormancy in breast cancer [14]. It was also suggested that LPA has a variety of biological activities involved in tumor initiation and progression, such as improved cell survival and angiogenesis [15]. Moreover, the key roles of LPA4 and LPA6 in developing angiogenesis were also addressed before [16]. Therefore, our study aims to verify our assumption whether miR-367 targeted LPA1 to affect ovarian cancer progression, so as to provide a potential approach to ovarian cancer treatment.

## Materials and methods

### Ethics statement

The experiment was authorized by the Ethics Committee of the Affiliated Suzhou Science & Technology Town Hospital of Nanjing Medical University and conducted in compliance with the *Declaration of Helsinki*. All individuals signed informed written consent documents. The experiments involving animals were performed complying with the *Guide for the Care and Use of Laboratory Animals*. Animal experiments were conducted according to the animal experiment system ethics guidelines approved by the Animal Management Committee of the Affiliated Suzhou Science & Technology Town Hospital of Nanjing Medical University.

### Microarray-based analysis

The miRNA expression microarray dataset GSE48485 and mRNA expression microarray data GSE66957 related to ovarian cancer were obtained from the Gene Expression Omnibus (GEO) database (https://www.ncbi.nlm.nih.gov/), including 5 cancer tissues and 5 adjacent normal tissues in GSE48485 dataset and 57 cancer tissues and 12 adjacent normal tissues in GSE66957 dataset. The threshold of |log Foldchange| > 1, *p* value < 0.05 was set to screen differentially expressed genes. Target mRNAs for significantly differential miRNAs, and the binding site map of miRNA and target gene were predicted by the StarBase database (http://starbase.sysu.edu.cn/index.php). The expression level of the predicted gene was obtained by performing the differential analysis on GSE66957 dataset.

### Clinical sample collection

We recruited 48 pairs of ovarian cancer and adjacent normal tissues (non-cancerous tissues verified by pathological examination) collected from ovarian cancer patients (aged 35–65 with a mean age of 48.83 ± 9.53 years old) underwent surgeries at Huzhou central Hospital from January 2014 to January 2016. Among these ovarian cancer patients, 18 were in stage I, 13 were in stage II, and 17 were in stage III [17]. According to histopathological grading criteria, high- and medium-(G1 + G2) (n = 28) and poor-differentiation (G3) (n = 20) of tumor were classified. Besides, cases of tumor diameter < 2 cm (n = 25) and ≥ 2 cm (n = 23) were assessed. Patients with other malignant tumors, severe infections, cognitive impairment, poor compliance or inability to understand the research process were excluded. The collected sample tissues were mainly divided into two parts. One part of the tissues was stored in a liquid nitrogen immediately for RNA and protein extraction. Another part of the tissues was fixed with paraformaldehyde and embedded in paraffin for subsequent experiments. The patients were followed up for 6–36 months with outpatient review and telephone follow-up, which was ended by June 2019. A total of 48 patients were followed up.

### Cell culture

Human ovarian cancer cell line A2780, CP70, SKOV3, and normal ovarian epithelial cell line IOSE80 were purchased from Beijing Beina Science and Technology Co., Ltd. (Beijing, China). (http://www.bncc.org.cn/default.htm). A2780, CP70, and IOSE80 cells were cultured in Dulbecco’s modified Eagle’s medium (DMEM)-H (Gibco; Thermo Fisher Scientific, USA) with 10% fetal bovine serum (FBS; Gibco). SKOV3 cells were cultured in RPMI-1640 medium (Gibco) with 10% FBS. All medium contained 100 U/mL penicillin, and all cell lines were cultured at 37 °C in 5% CO_2_ and then subcultured. The human umbilical vein endothelial cells (HUVECs) were from ATCC (ATCC^®^ CRL-1730).

### Cell transfection

Cells were seeded into a 24-well plate (2.5 × 10^5^ cells/well) and transfected after the density reached at 60–70%. Ovarian cancer cells were grouped as mimic NC, miR-367 mimic, inhibitor NC, miR-367 inhibitor, si-NC, siRNA targeting LPA1 (si-LPA1) (si-LPA1-1: GGAGGAUGUCUGAGAGAAAGA; si-LPA1-2: CCAUGUUGUUAACUAUUUAGG; si-LPA1-3: CGAUCUGAUCAGCAAACAAGA), and si-LPA1 + miR-367 mimic groups. miR-367 mimic, miR-367 inhibitor, and si-LPA1 were purchased from Ribobio (Guangzhou, China). Cells were transfected following the instructions of Lipofectamine™ (Invitrogen, Carlsbad, USA).

### RT-qPCR

Total RNA was extracted using Trizol (15596026, Invitrogen), and then reversely transcribed into complementary DNA (cDNA) by PrimeScript RT reagent Kit (RR047A, Takara, Tokyo, Japan). For each sample, 1000 ng RNA was reversely transcribed into 20 μL cDNA, and 2 μL cDNA was used for subsequent PCR operation. Primers for miR-367, LPA1, MCM2, MMP2, MMP9, VEGF, U6, and GADPH were designed and synthesized by Shanghai Sangon Biotech (Shanghai, China) (Table 1). The reaction solution was taken for real-time fluorescence quantitative PCR operation by ABI 7500 real time quantitative PCR instrument (7500, ABI, Perkin-Elmer, Applied Biosystems, Foster City, CA, USA). The 2^−△△Ct^ method was used to calculate the expression levels of miR-367, LPA1, MCM2, MMP2, MMP9, and VEGF in cells.

**Table 1:** Primer sequences for RT-qPCR

Gene	Forward primer (5′-3′)	Reverse primer (5′-3′)
miR-367	ACTGCAAGAAACGGTTTTCCC	GGCGCGGAACACTGAGATGT
LPA1	ATCTTTGGCTATGTTCGCCA	TTGCTGTGAACTCCAGCCA
MCM2	CACATCGAGTCCATGATCC	CAAAAGTCTTGCGCATGCT
MMP2	TTTGCTCGGGCCTTAAAAGTAT	CCATCAAACGGGTATCCATCTC
MMP9	CGGACCCGAAGCGGACAT	GGGGCACCATTTGAGTTT
VEGF	CATGAACTTTCTGCTGTCTTGG	CCTGGTGAGAGATCTGGTTCC
GADPH	ACCACAGTCCATGCCATCAC	TTACCACCCTGTTGCTGTA
U6	GCTTCGGCAGCACATATACTAAAA	CGCTTCACGAATTTGCGTGTCAT

*RT-qPCR* reverse transcription quantitative polymerase chain reaction, *miR-367* microRNA-367, *LPA1* lysophosphatidic acid receptor-1, *MCM2* minichromosome maintenance 2, *MMP2* matrix metalloproteinase 2, *MMP9* matrix metalloproteinase 9, *VEGF* vascular endothelial growth factor, *GAPDH* glyceraldehyde-3-phosphate dehydrogenase

### Immunoblotting

Total protein was harvested using an assay kit (Beyotime Biotechnology, Shanghai, China). Bio-Rad DC Protein Assay Kit (Guangzhou Ewell Bio-technology Co., Ltd., Guangdong, China) was used for protein quantification. Each sample was added with sodium dodecyl sulfate (SDS) loading buffer, boiled for 10 min in boiling water, and 20 μg protein sample was applied to a 10% SDS-polyacrylamide gel. Then the protein was transferred onto polyvinylidene fluoride membrane and immersed in 1 × Tris-Buffered Saline Tween-20 (TBST) containing 5% skimmed milk powder to block non-specific binding sites. The membrane was then incubated overnight at 4 °C with diluted primary antibody, i.e. one of the rabbit antibodies LPA1 (R&D system, Minneapolis, MN, USA, AF9963, 1:2000), MCM2 (R&D system, AF5778, 1:2000), MMP2 (R&D system, AF902, 1:2000), MMP9 (R&D system, AF909, 1:2000), VEGF (R&D system, AF-493-NA, 1:2000), and GAPDH (R&D system, AF5718, 1:2000). Then the membrane was incubated with secondary goat anti-rabbit anti-immunoglobulin G (IgG) (R&D system, AB-105-C, 1:20,000). Exposure was carried out with an enhanced chemiluminescence. Gray value of each protein was determined by Image J software (NIH free software, Bethesda, MD, USA). The original immunoblotting bands are shown in Additional file 1.

### 5-ethynyl-2′-deoxyuridine (EdU) assay

A2780 cell proliferation experiments were performed using the EdU assay kit (CA1170, Beijing Solarbio Science & Technology Co., Ltd., Beijing, China). Cells were seeded in 96-well plates with 1 × 10^4^ cells/well. Then 100 μL of 50 μM EdU medium was added to each well. The cells were fixed with 40 g/mL paraformaldehyde for 20 min, incubated with 2 mg/mL glycine for 10 min, and washed twice by phosphate-buffered saline (PBS). Each well was added with 100 μL of penetrant (0.5% Triton X-100 in PBS) (T8200, Beijing Solarbio Science & Technology Co., Ltd.), shaken on a bleaching shaker for 10 min, and then added with 100 μL of Apollo staining solution for incubation for 30 min in the dark. Next, cells were added with Hoechst33342 reaction solution to incubate at room temperature for 30 min, washed twice with 0.5% Triton X, and observed under an inverted fluorescence microscope. Image-pro plus (IPP) 6.0 professional image analysis software (VersionX, Media Cybernetics, Silver Springs, MD, USA) was used to count the number of cells.

### Transwell assay

3 × 10^4^ A2780 cells were added in the apical chamber with 200 μL of serum-free medium, and 500 μL of fresh medium containing 10% FBS was supplemented in the basolateral chamber. The insert was coated with 200 mg/mL Matrigel and the cells were incubated for 24 h for invasion assay. After 48 h, the cells invaded in basolateral chamber were stained with 0.1% crystal violet. Images of invaded A2780 cells were observed and collected by a phase contrast microscope.

### Dual luciferase reporter gene assay

Bioinformatics website was used to predict binding site of miR-367 and LPA1 and to obtain fragment sequences containing the site of action. The 3′ UTR region of LPA1 was cloned and amplified into a pmirGLO (E1330, Promega, Madison, WI, USA) luciferase vector and named as pWt-LPA (CUUGGUAGCCACACCUGCAAUG). The pMut-LPA vector (CUUGGUAGCCACACCGACGUCG) was also constructed. Then the pRL-TK vector expressing Renilla luciferase (E2241, Promega, Madison, WI, USA), mimic NC, and miR-367 mimic were co-transfected with luciferase reporter vectors pWt-LPA and pMut-LPA respectively into ovarian cancer cell line A2780. The luminescence intensity was monitored by the Dual Luciferase Reporter Gene Assay Kit (GM-040502A, Qiancheng Bio, shanghai, China) at 560 nm (firefly relative luciferase units (RLU)) and 482 nm (renilla RLU), and the firefly RLU/renilla RLU ratio was measured to determine the binding.

### RNA immunoprecipitation (RIP)

Bindings of miR-367 and LPA1 to Ago2 protein were assessed according to Magna RIP RNA-binding protein immunoprecipitation kit (Merck Millipore, Billerica, USA). Cells were washed with pre-cooled PBS and the supernatant was discarded. The cells were lysed with an equal volume of RIPA lysate for 5 min in ice bath, centrifuged at 14,000 rpm for 10 min at 4 °C, followed by the removal of supernatant. A part of the cell extract was taken out as an input, and the rest was incubated with the antibody for coprecipitation. Specifically, 50 μL of magnetic beads for each coprecipitation reaction system was washed and resuspended in 100 μL of RIP wash buffer, and 5 μg of antibody was added for combination. The magnetic bead-antibody complex was washed and resuspended in 900 μL of RIP wash buffer, and 100 μL of cell extract was added. Next, the mixture was incubated, and the sample was placed on the magnetic base to collect the magnetic bead-protein complex. The sample and input were separately treated by proteinase K to extract RNA for subsequent PCR detection. IgG was used as NC.

### Tube formation assay

Tube formation assay was conducted on HUVECs under the treatment of different ovarian cancer cell culture medium. HUVECs were cultured in DMEM medium containing 10% FBS. Ovarian cancer cells A2780 were cultured in DMEM-H with 10% FBS medium at 37 °C. The ovarian cancer cells were transfected, and the culture medium was collected 48 h later. Tumor conditioned medium was prepared by tumor supernatant: DMEM medium: FBS at a ratio of 4:5:1. A total of 50 μL Matrigel glue was added to each well of a 96-well plate and gelled at 37 °C for 30 min. Then cells were added with prepared tumor conditioned medium and HUVECs suspension and cultured. The operation was replicated three times in each group, with 4 fields of view taken for the observation under a phase contrast microscope. The number of small tubes was counted and photographed.

### Chick chorioallantois membrane (CAM) assay

Seventy healthy chicken embryos were randomly injected with miR-367 mimic, miR-367 inhibitor, si-LPA1, miR-367 inhibitor and si-LPA1 or their negative control (mimic NC, inhibitor NC, and si-NC). 1 × 10^7^ A2780 cells in the exponential growth phase was inoculated on the 10 days well-developed fertilized CAM using chicken fenestration. After inoculation, the chicken embryos were incubated for 5 days. The number of CAM vessels (number, N), area of blood vessel (area, A), ratio of blood vessel area (VA/A), tissue surface, and tissue area (tissue, T) of each group were detected by image analysis, and blood vessel and tissue changes were observed.

### Xenografts in nude mouse

Twenty female BALB/C nude mice were obtained from Guangdong Medical Laboratory Animal Center (Guangdong, China). Mice were injected with cells transfected with si-NC and si-LPA1 (constructed by Shanghai Sangon Biotech) respectively (10 in each group). The above stably transfected cells were subcutaneously injected into the armpits of female BALB/C nude mice (4–6 weeks old, 18–22 g) (1 × 10^7^ cells/each mouse). Tumor growth was monitored every 3 days by measuring the width (W) and length (L) with a caliper, and the volume of the tumor (V) was calculated using the formula V = (W^2^ × L)/2. Four weeks after the injection, the mice were euthanized and the tumor weight was measured.

### Immunohistochemistry

Specimens were fixed with 10% formaldehyde and embedded in paraffin. Then 4 μm serial section was cut. Tissue sections were placed in a 60 °C incubator for 1 h, dewaxed by xylene, dehydrated with gradient alcohol, and incubated in 3% H_2_O_2_ (Sigma-Aldrich, St. Louis, MO, USA). Then tissue sections were washed with PBS, placed in 0.01 M citrate buffer, boiled at 95 °C for 20 min, cooled to room temperature, rinsed with PBS, and blocked with normal goat serum working solution with 37 °C for 10 min. Tissue sections were incubated with anti-rabbit CD34 (R&D system, AF4117, 1:100) and LPA1 (R&D system, AF9963, 1:100) at 4 °C for 12 h. After washing with PBS, the corresponding biotin-labeled secondary antibody IgG goat anti-rabbit (R&D system, AB-105-C, 1:20,000) was added and kept for 10 min. Next, horseradish peroxidase-labeled streptavidin working solution (S-A/HRP) was added and kept for 10 min. Tissue sections were developed with diaminobenzidine (DAB) and stored in the dark for 8 min. Then cells were washed with tap water, stained with hematoxylin, dehydrated, transparentized, blocked, and observed by light microscopy. Positive cell counts were performed using Japanese Nikon image analysis software, and three equal-area non-repetitive fields (× 200) were selected for each slice to calculate the number of positive cells. ABCF2 (positive staining is greater than 25% of cells) and brown or brownish yellow particles appear in the cytoplasm were regarded as the criteria for immunohistochemistry. Positive expression rate = number of positive cases/total number of cases.

### Statistical analysis

Data statistical analyses were processed using Statistic Package for Social Science (SPSS) 21.0 software (IBM Corp. Armonk, NY, USA). Measurement data were presented as mean ± standard deviation. Paired data between two groups were compared using paired *t* test, while unpaired data were analyzed using unpaired t-test. Comparisons among multiple groups were conducted by one-way analysis of variance (ANOVA) followed by Tukey’s post hoc test. Chi-square test was used to analyze the number of high and low expression cases. Patients’ survival rate was analyzed using the Kaplan–Meier method, while the difference of miR-367 expression was assessed by logrank test. The correlation between miR-367 and LPA1 was evaluated by Pearson’ correlation coefficient. The xenograft tumors in nude mice at different time points were observed and analyzed by repeated measures ANOVA. Differences were considered statistically significant when *p *< 0.05.

## Results

### miR-367 might participate in ovarian cancer progression by regulating LPA1

**Fig. 1:**
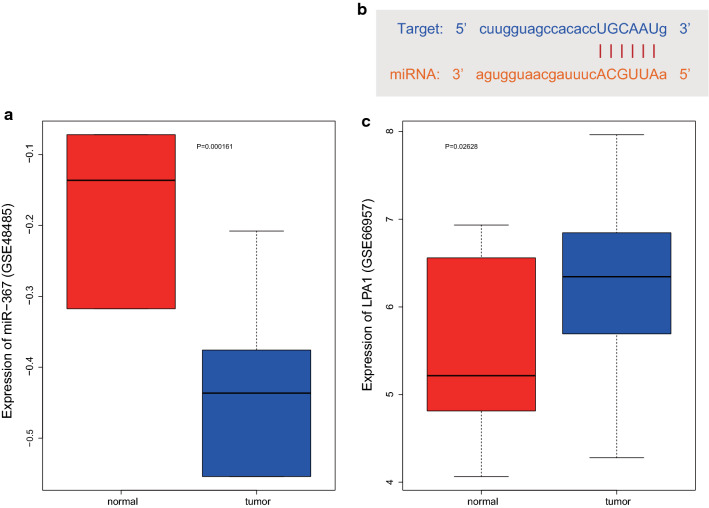
miR-367 is poorly expressed but LPA1 is highly expressed in ovarian cancer cells. **a** Differential expression of miR-367 in cancer tissue samples and adjacent normal tissue samples in ovarian cancer microarray dataset GSE48485. **b** Binding site map of miR-367 to the target gene LPA1 in the StarBase database. **c** Box diagram of differential expression of LPA1 gene in cancer tissue samples and adjacent normal tissue samples in ovarian cancer microarray dataset GSE66957

To screen out the miRNA involved in ovarian cancer, the miRNA expression microarray dataset GSE48485 and mRNA expression microarray dataset GSE66957 of ovarian cancer were obtained from the GEO database. Following differential analysis of the gene expression data GSE48485, miR-367 was identified with the highest fold change and the lowest *p* value and was selected for further analysis. Result of GSE48485 differential analysis indicated that miR-367 was poorly expressed in ovarian cancer (Fig. 1a). The target mRNA of miR-367 was predicted by StarBase database, which found that there were binding sites of miR-367 on LPA1 (Fig. 1b). Meanwhile, LPA1 was found to be highly expressed in cancer metastatic cell lines, and LPA1 overexpression could promote the invasion and migration of ovarian cancer cells [18]. To further validate the expression of LPA1, we performed a differential analysis of GSE66957 and obtained high expression of LPA1 in ovarian cancer (Fig. 1c). It is also reported that LPA1 could promote tumor angiogenesis [19]. These results suggested that miR-367 might affect the progression of ovarian cancer cells by regulating LPA1.

### miR-367 is poorly expressed in ovarian cancer cells

**Fig. 2:**
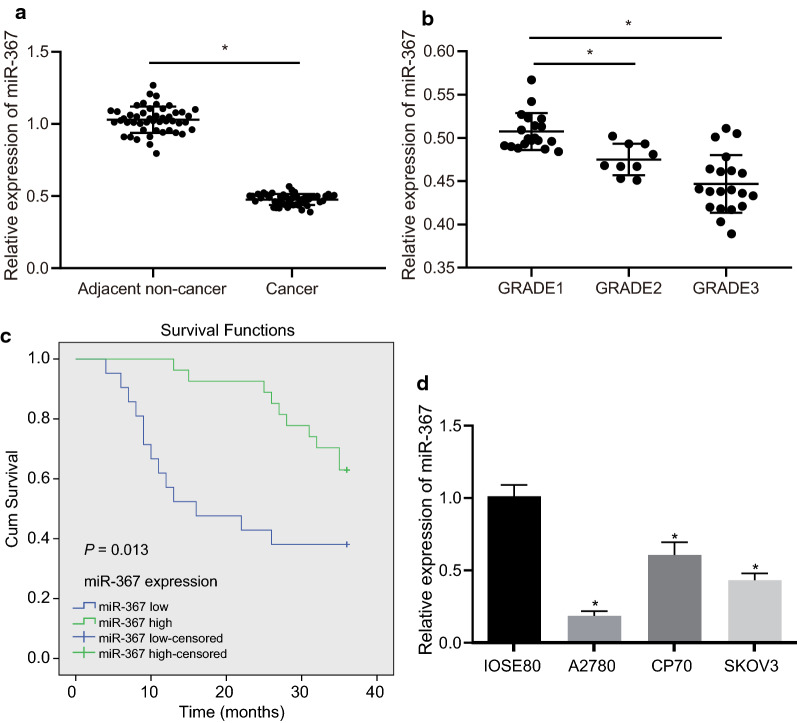
Low expression of miR-367 in ovarian cancer cells. **a** Relative expression levels of miR-367 in ovarian cancer tissues and adjacent normal tissues measured by RT-qPCR. **b** RT-qPCR detection on relative expression of miR-367 in different differentiation degree of ovarian cancer. **c** Prognostic analysis based on the average expression level of miR-367 in ovarian cancer tissues. **d** RT-qPCR detection on relative expression levels of miR-367 in normal ovarian epithelial cell lines and ovarian cancer cell lines. Measurement data were expressed as mean ± standard deviation. Data in compliance with normal distribution and homogeneity between two groups were compared using paired *t* test. Comparisons among multiple groups were conducted by one-way ANOVA with Tukey’s post hoc tested. *p < 0.05 indicated significant difference. The experiment was repeated three times independently. The number of experimental tissues was 48

To clarify the expression of miR-367 in ovarian cancer, we first detected the expression of miR-367 in 48 pairs of ovarian cancer and adjacent normal tissues by RT-qPCR (Fig. 2a). The results proved that the miR-367 expression in ovarian cancer tissues was lower than that in adjacent normal tissues (*p* < 0.05). Moreover, the level of miR-367 in ovarian cancer cells gradually decreased from grade 1 (well differentiated) to grade 3 (poorly differentiated) (*p* < 0.05) (Fig. 2b). At the same time, the correlation between the expression of miR-367 and the clinicopathological features of ovarian cancer was tested (Table 2). Results revealed that the expression of miR-367 was higher in tumors with the length < 2 cm than in tumors with the length ≥ 2 cm (*p* < 0.05). The miR-367 expression in the high- and medium-differentiation group was higher than in the poor differentiation group, it was lower in the lymph node metastasis group than non-lymph node metastasis group (all *p* < 0.05). Subsequently, the average expression of miR-367 in 48 ovarian cancer patients was calculated for Cut-off value, which was used for prognostic analysis. The results revealed that patients with low expression of miR-367 had lower survival rate (38.10%) than those with high expression of miR-367 (62.96%) with a total survival rate of 52.08% (Fig. 2c). Further cellular experiments also showed that miR-367 was downregulated in cancer cell lines A2780, CP70, and SKOV3 compared with the normal ovarian epithelial cell line IOSE80, among which miR-367 showed the lowest expression in the ovarian cancer cell line A2780 (*p* < 0.05) (Fig. 2d). Thus, cell line A2780 was selected for transfection and subsequent related experiments. The results above proved that miR-367 was poorly expressed in ovarian cancer cells.

**Table 2:** Correlation between miR-367 level in ovarian cancer tissues and clinicopathological features

Clinicopathological features	Cases (n)	High expression	Low expression	χ^2^	*P*
Age (years)				0.117	0.732
< 45	17	9	8		
≥ 45	31	18	13		
Tumor size (cm)				8.27	0.004
< 2	25	19	6		
≥ 2	23	8	15		
Histopathological grading				18.31	< 0.001
G1 + G2	28	23	5		
G3	20	4	16		
Clinical staging				21.17	< 0.001
I + II	31	25	6		
III	17	2	15		
Lymph node metastasis				12.1	0.001
Without	35	25	10		
With	13	2	11		
Histotype				9.48	0.024
Serosity	19	6	13		
Endometrioid	12	7	5		
Mucinous	10	8	2		
Clear cell	7	6	1		

### Overexpression of miR-367 inhibits proliferation, invasion, and angiogenesis of ovarian cancer cells

**Fig. 3:**
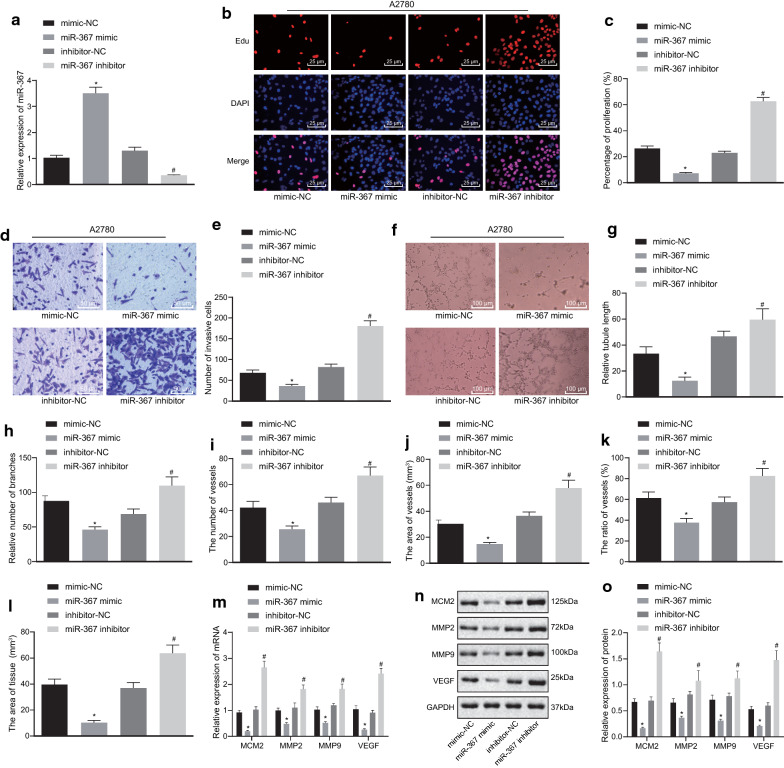
Overexpression of miR-367 suppresses proliferation, invasion, and angiogenesis of ovarian cancer A2780 cells. **a** RT-qPCR detection on miR-367 vectors transfection efficiency. **b** The proliferation of A2780 cells (scale bar = 25 μm) measured by EdU assay. **c** Ovarian cancer cell proliferation rate statistics. **d** The invasive ability of A2780 cells detected by Transwell assay (scale bar = 50 μm). **e** Statistical graph of A2780 cell invasion and relative cell number. **f** Representative image of tube formation (scale bar = 100 μm). **g** Statistics of relative length of blood vessels. **h** Relative number of blood vessel branches. **i** CAM blood flow statistics. **j** CAM blood vessel area statistics. **k** CAM blood vessel area ratio chart. **l** CAM tissue area chart. **m** RT-qPCR results of mRNA expression levels of related factors. **n** Western blot analysis of the protein expression level of related factors. **o** Protein expression levels of related factors. Measurement data were expressed as mean ± standard deviation. Comparisons among multiple groups were conducted by one-way ANOVA followed by Tukey’s post hoc test. *p < 0.05 vs. NC; ^#^*p* < 0.05 vs. Inhibitor NC. The experiment was repeated three times independently

To investigate whether the altered expression of miR-367 affected the biological function of ovarian cancer cells, the ovarian cancer cell line A2780 was selected, transfected, and then miR-367 expression was detected. Compared with the A2780 cells transfected with mimic NC, the expression of miR-367 was increased in the A2780 cells transfected with miR-367 mimic (*p* < 0.05), and the expression of miR-367 was decreased in the A2780 cells transfected with miR-367 inhibitor compared with the cells transfected with inhibitor NC (*p *< 0.05), suggesting successful transfection of the vectors (Fig. 3a). Subsequently, the proliferation of A2780 cells was detected by EdU assay. The results showed that the proliferation ability of A2780 cells treated with miR-367 mimic was lower than that of A2780 cells treated with mimic NC (*p *< 0.05), while proliferation ability of the A2780 cell treated with miR-367 inhibitor was higher than that of the A2780 cells treated with inhibitor NC (*p *< 0.05) (Fig. 3b, c). Transwell assay for cell invasion ability proved that cell invasion ability was reduced with miR-367 mimic treatment in contrast to mimic NC treatment (*p *< 0.05), while ability was enhanced with miR-367 inhibitor treatment in contrast to inhibitor NC treatment (*p *< 0.05) (Fig. 3d, e). The results of tube formation assay showing tumor-induced HUVECs angiogenesis indicated that the angiogenic ability of A2780 cells treated with miR-367 mimic was lower than that of cells treated with mimic NC (*p *< 0.05), while angiogenic ability of A2780 cells increased after suppressed miR-367 (Fig. 3f–h). At the same time, the results of CAM assay on the growth of blood vessels and tissues demonstrated that the number, area, and area ratio of blood vessel and tissue area in the miR-367 mimic-treated A2780 cells were smaller than those in the mimic-NC-treated cells (*p *< 0.05), while were larger in the miR-367 inhibitor-treated A2780 cells than those in the mimic-NC-treated A2780 cells from CAM assay (*p *< 0.05) (Fig. 3i–l).

In addition, we determined the mRNA and protein level of proliferation-related factor MCM2, invasion-related factor MMP2, MMP9, and angiogenesis-related factor VEGF by RT-qPCR and Immunoblotting. The results (Fig. 3m–o) displayed that compared with the A2780 cell transfected with mimic NC and inhibitor NC, the expression of MCM2, MMP2, MMP9, and VEGF in the A2780 cells transfected with miR-367 mimic were inhibited (all *p *< 0.05), while those in the A2780 cells transfected with miR-367 inhibitor were increased (all *p *< 0.05).

Taken together, the above results indicated that overexpression of miR-367 reduced the proliferative and invasive ability of ovarian cancer cells and inhibited tumor-induced HUVECs angiogenesis.

### miR-367 downregulates the expression of LPA1

**Fig. 4:**
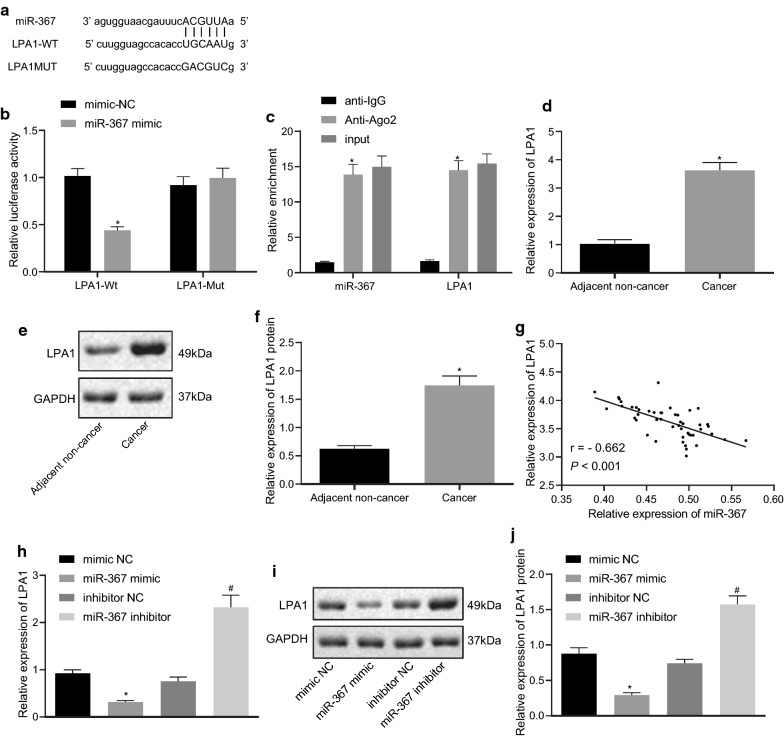
miR-367 downregulates LPA1 expression. **a** Bioinformatics prediction of binding sites for miR-367 and LPA1. **b** Luminescence activity of LPA1 by dual luciferase reporter gene assay. **c** RIP assay detection of the binding of miR-367 and LPA1 to Ago2. **d** Detection of relative expression of LPA1 in cancer tissues and adjacent normal tissues by RT-qPCR. **e** Analysis of protein level of LPA1 in cancer and adjacent normal tissues by western blot analysis. **f** Statistical expression of protein level of LPA1. **g** Correlation analysis between miR-367 and LPA1. **h** RT-qPCR results of the relative expression level of LPA1 in each group of cells. **i** Western blot analysis of LPA1 protein levels in each group of cells. **j** Statistical protein expression level of LPA1. Measurement data were expressed as mean ± standard deviation. Data in compliance with normal distribution and homogeneity between two groups were compared using *t* test. Comparisons among multiple groups were conducted by one-way ANOVA followed by Tukey’s post hoc test. *p < 0.05 vs. normal tissues or cells; ^#^*p* < 0.05 vs. Inhibitor NC The experiment was repeated three times independently

To study the targeting relationship of miR-367 in ovarian cancer cells, firstly, we found that there were binding sites for miR-367 and LPA1 through bioinformatics website analysis (Fig. 4a). Then, the results of dual luciferase reporter assay revealed that the luciferase activity of the LPA1 Wt 3′UTR was inhibited by miR-367 compared to the cell treated with mimic-NC (*p *< 0.05), while the luciferase activity of the LPA1 Mut 3′UTR showed no significant change (*p *> 0.05) (Fig. 4b). The results of RIP experiments showed that the bound miR-367 and LPA1 were higher in the Ago2 group compared with the IgG group, suggesting that miR-367 specifically bound to the 3′UTR region of LPA1 and downregulated LPA1 expression at the post-transcriptional level (Fig. 4c). Then, RT-qPCR and Immunoblotting were used to measure the expression of LPA1 in ovarian cancer and adjacent normal tissues. The result demonstrated that LPA1 was highly expressed in ovarian cancer tissues (Fig. 4d–f). Moreover, correlation analysis showed that miR-367 was negatively correlated with LPA1 expression (Fig. 4g). Subsequently, the mRNA and protein level of LPA1 in each group was detected by RT-qPCR and western blot analysis through interfering with the expression of miR-367 (Fig. 4h–j). The results of the analysis proved that the expression of LPA1 in the miR-367 mimic group were lower than those in the mimic-NC group (*p *< 0.05). Compared with the inhibitor-NC treatment, the mRNA and protein expression levels of LPA1 in the cells treated with miR-367 inhibitor was increased (*p *< 0.05). The above results indicated that LPA1 was highly expressed in ovarian cancer and LPA1 was a direct target of miR-367.

### Low expression of miR-367 upregulates LPA1 expression to promote ovarian cancer cells proliferation, invasion, and angiogenesis

**Fig. 5:**
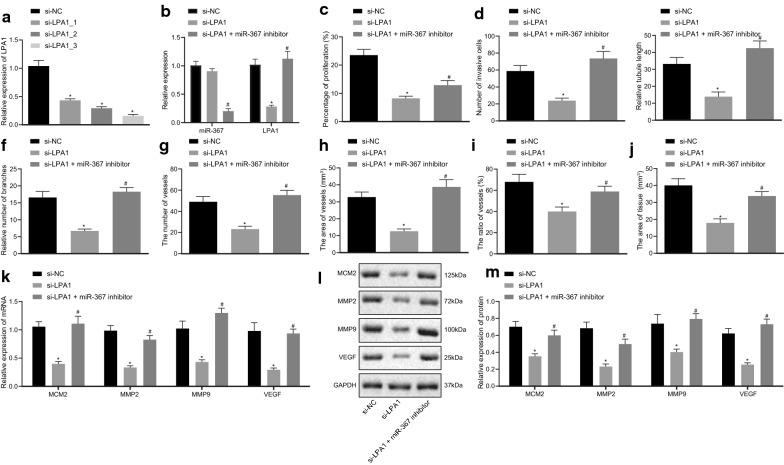
Low expression of miR-367 upregulates the expression of LPA1 to promote proliferation, invasion, and angiogenesis of ovarian cancer A2780 cells. **a** RT-qPCR analysis results of the efficiency of LPA1 silencing by appropriate siRNAs. **b** miR-367 and LPA1 expression in cells determined by using RT-qPCR. **c** A2780 cell proliferation ability detected by Transwell assay (scale bar = 25 μm). **d** Invasion ability of A2780 cells measured by Transwell assay (scale bar = 50 μm). **e** Relative length of blood vessels. **f** Relative number of blood vessel branches. **g**–**j** Statistics of CAM blood vessel number, area, vessel area ratio and tissues area. **k** RT-qPCR detection of mRNA levels of related factors. **l** Western blot analysis of protein levels of related factors. **m** Statistics of protein expression levels of related factors. Measurement data were expressed as mean ± standard deviation. Data in compliance with normal distribution and homogeneity among multiple groups were conducted by one-way ANOVA followed by Tukey’s post hoc test. *p < 0.05 vs. NC. ^#^*p *< 0.05 vs. cells treated with si-LPA1. The experiment was repeated three times independently

In order to further study the effect of LPA1 on the biological function of ovarian cancer cells, we designed three si-LPA1s, and screened the one with the highest interference efficiency of si-LPA1_3 after transfection (Fig. 5a). At the same time, RT-qPCR found that the expression of miR-367 had no change and the expression of LPA1 was reduced in the si-LPA1 group compared with the si-NC group, while compared with the si-LPA1 group, the expression of miR-367 was suppressed and the expression of LPA1 was increased in the si-LPA1 + miR-367 inhibitor group (Fig. 5b). The proliferation of A2780 cells was examined by EdU assay, which showed that the proliferation ability of cells in si-LPA1-treated A2780 cells was lower than that in si-NC-treated cells, while si-LPA1 + miR-367 inhibitor cotreated A2780 cells exhibited higher proliferation ability than that treated with si-LPA1 alone (*p *< 0.05) (Fig. 5c). The results of Transwell assay detecting A2780 cell invasion ability proved that the invasive ability of the A2780 cells transfected with si-LPA1 was decreased by contrast in the A2780 cells transfected with si-NC, while in contrast to the A2780 cells treated with si-LPA1, the invasive ability of miR-367 inhibitor and si-LPA1 cotreated A2780 cells was improved (*p *< 0.05) (Fig. 5d). The results of tube formation assay (Fig. 5e) and CAM assay (Fig. 5f–j) revealed that compared with the si-NC-treated A2780 cells, the angiogenic ability of the si-LPA1-treated A2780 cells was reduced (*p *< 0.05), while the angiogenic capacity of miR-367 inhibitor and si-LPA1 cotreated A2780 cells was increased in contrast to the si-LPA1 treated A2780 cells (*p *< 0.05).

At the same time, RT-qPCR and immunoblotting were performed to detect the mRNA and protein levels of MCM2, MMP2, MMP9, and VEGF. The results exhibited that compared with the A2780 cells transfected with si-NC, the expression of MCM2, MMP2, MMP9, and VEGF in A2780 cells transfected with si-LPA1 were decreased (*p *< 0.05), while those were higher in A2780 cells cotreated with miR-367 inhibitor and si-LPA1 than those treated with si-LPA1 alone (*p *< 0.05) (Fig. 5k–m). These results suggested that low expression of miR-367 raised LPA1 expression to improve proliferation, invasion, and angiogenesis of ovarian cancer cells.

### Low expression of LPA1 inhibits tumor formation ability of ovarian cancer in nude mice

**Fig. 6:**
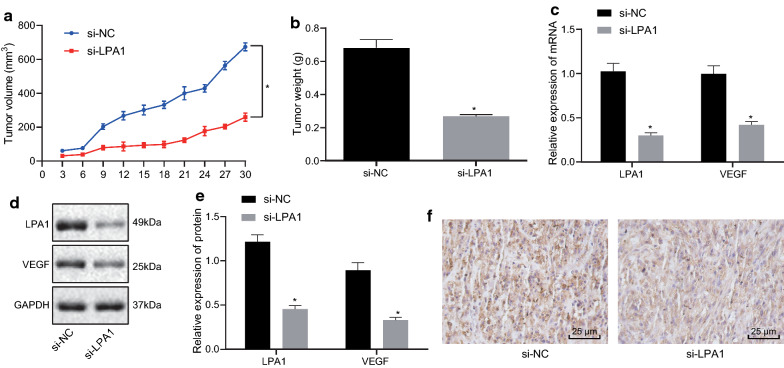
Low expression of LPA1 inhibits tumorigenic ability of ovarian cancer in nude mice. **a** Volume of mice tumor. **b** Weight of mice tumor. **c** RT-qPCR detection results of mRNA levels of related factors in tumor tissues. **d** Western blot analysis of the protein levels of related factors in tumor tissues. **e** Statistical graph of protein levels of related factors. **f** Immunohistochemistry for detecting CD34 expression (200×). Measurement data were expressed as mean ± standard deviation. Data in compliance with normal distribution and homogeneity between two groups were compared using *t* test. Comparisons among multiple groups were conducted by one-way ANOVA followed by Tukey’s post hoc test. Statistics of tumor volume at different time points was analyzed using repeated measures ANOVA. *p < 0.05 vs. normal tissues or cells. The experiment was repeated three times independently

To investigate the effect of LPA1 on xenograft tumorigenesis in nude mice, cells stably transfected with si-LPA1 and si-NC were injected into nude mice. Tumor volume was measured after injection, which showed that the tumor volume gradually increased along injection time (Fig. 6a, b). During the same time, the average of tumor volume and weight of mice in the si-LPA1 group was decreased compared with mice in the si-NC group (*p *< 0.05). Meanwhile, RT-qPCR and Immunoblotting were used to detect the mRNA and protein levels of LPA1 and VEGF in tumor. The result proved that by contrast in the mice injected with si-NC transfected cells, the level of LPA1 and VEGF in the mice injected with si-LPA1 transfected cells were decreased (all *p *< 0.05) (Fig. 6c–e). In addition, immunohistochemistry was used to detect microvascular density marker CD34 expression, and the result revealed that the CD34-labeled microvessel density in the mice injected with si-LPA1 transfected cells was distinctly lower than that in the mice injected with si-NC transfected cells (*p *< 0.05) (Fig. 6f). The above results indicated that low expression of LPA1 reduced the tumorigenic ability of ovarian cancer cells and inhibited the angiogenic ability of ovarian cancer cells.

## Discussion

Plasma miRNAs serve as biomarkers for ovarian cancer prognosis and diagnosis [20]. It is reported that though many hurdles need to be overcome, miRNA therapy might work as a powerful treatment method to prevent and cure ovarian cancer [21]. For example, overexpression of miR-155 could prevent tumorigenesis in human ovarian cancer by downregulating CLDN1 [22]. We aimed to investigate the mechanism by which miR-367 involved in ovarian cancer development. Collectively, the data of this study revealed that miR-367 suppressed the development of ovarian cancer by downregulating the expression of LPA1.

The first finding of this study was that miR-367 was poorly-expressed in ovarian cancer cells, and overexpression miR-367 inhibited proliferation, invasion, and angiogenesis of ovarian cancer cells. Firstly, MCM2 positive cells represents the proliferating breast cancer cells [23]. Another study also reported that the high expression of MCM2 in serrated polyps showed abnormal cell proliferation [24]. Moreover, MMP2 and MMP9, as proteolytic enzymes, are involved in the degradation of extracellular matrices, which play a crucial role in tumor invasion and metastasis [25]. In addition, MMPs such as MMP2 and MMP9 could be prognostic biomarkers for ovarian cancer [26]. It is also revealed that the VEGF receptor endocytosis regulates vessel growth in angiogenesis [27]. Our results showed that miR-367 inhibited the expression of MCM2, MMP2, MMP9, and VEGF, suggesting that miR-367 may repress tumor cell proliferation, migration, invasion, and angiogenesis in ovarian cancer.

We further found that LPA1 was highly expressed in ovarian cancer tissues and cells, and low expression of LPA1 reduced tumorigenic and angiogenic ability of ovarian cancer cells. LPA was reported to regulate pathological processes such as embryonic development, angiogenesis, and tumor progression [12]. Besides, LPA is an autocrine growth signal, which is significant for the occurrence of ovarian cancer [28] influencing the pathology of human ovarian cancer [29]. Our experiments demonstrated that LPA1 promoted angiogenesis and the ovarian cancer development.

Subsequently, we found that miR-367 targeted LPA1 expression and overexpression of miR-367 downregulated LPA1 expression to repress proliferation, invasion, and angiogenesis of ovarian cancer cells, thereby inhibiting ovarian cancer progression. Consistent with our work, previous studies also presented the targeting relation between miRNAs and other genes in ovarian cancer. For example, miR-17 suppresses peritoneal metastasis in ovarian cancer through ITGA5 and ITGB1 [30]. Similarly, miR-320 repressed oncogenicity of ovarian cancer by targeting TWIST1 expression [31]. However, to our best knowledge, this is the first report that revealed the targeting relationship between miR-367 and LPA1 and the mechanism in ovarian cancer.

## Conclusion

**Fig. 7:**
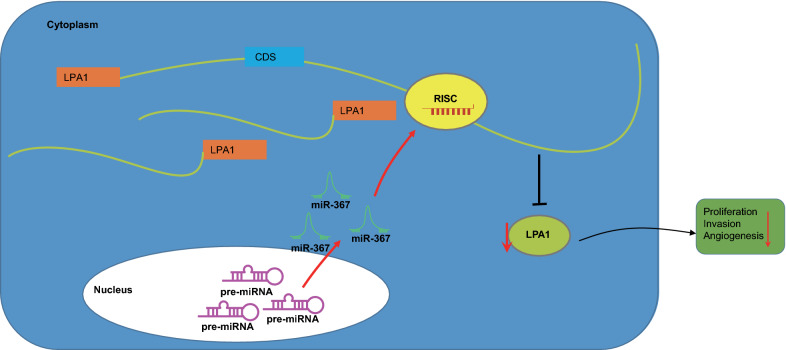
The regulatory mechanism map. Overexpressed miR-367 could reduce the expression of LPA1 and inhibit the proliferation and invasion of cancer cells, and the process of tumor-induced angiogenesis, hereby repressing ovarian cancer development

In conclusion, overexpression of miR-367 downregulated the expression of LPA1 in order to inhibit the proliferation and invasion of cancer cells and the process of tumor-induced angiogenesis, thus repressing ovarian cancer development (Fig. 7). Therefore, miR-367 might serve as a potential strategy for the ovarian cancer treatment. However, there are still several deficiencies remain in our study. For example, the study population was small. To overcome it, a diverse group of patients should be added into support our findings. For another thing, our study has not been verified in clinical trials. Therefore, there are many future experiments could be conducted to ensure the accuracy of our research results.

## Supplementary information

**Additional file 1** The original immunoblotting bands.

## Data Availability

The datasets generated/analysed during the current study are available.

## Abbreviations

miRNAs: MicroRNAs
LPA1: Lysophosphatidic acid receptor-1
CAM: Chick chorioallantois membrane
HUVECs: Human umbilical vein endothelial cells
DMEM: Dulbecco’s modified Eagle’s medium
FBS: Fetal bovine serum
RT-qPCR: Reverse transcription quantitative polymerase chain reaction
SDS: Sodium dodecyl sulfate
TBST: Tris-Buffered Saline Tween-20
EdU: 5-Ethynyl-2′-deoxyuridine
IPP: Image-pro plus
RLU: Relative luciferase units
RIP: RNA immunoprecipitation
DAB: Diaminobenzidine
SPSS: Statistic Package for Social Science
ANOVA: Analysis of variance
